# Updates on C3 Glomerulopathy in Kidney Transplantation: Pathogenesis and Treatment Options

**DOI:** 10.3390/ijms25126508

**Published:** 2024-06-13

**Authors:** Giulia Bartoli, Andrea Dello Strologo, Giuseppe Grandaliano, Francesco Pesce

**Affiliations:** 1 Department of Translational Medicine and Surgery, Università Cattolica dl Sacro Cuore, 00168 Rome, Italy; giulia23bartoli@gmail.com (G.B.); andreadellostrologo@gmail.com (A.D.S.); giuseppe.grandaliano@unicatt.it (G.G.); 2 Nephrology, Dialysis and Transplantation Unit, Fondazione Policlinico Universitario A. Gemelli IRCCS, 00168 Rome, Italy; 3 Division of Renal Medicine, “Ospedale Isola Tiberina—Gemelli Isola”, 00186 Rome, Italy

**Keywords:** kidney transplantation, C3 glomerulopathy, infection, monoclonal gammopathy, treatment

## Abstract

C3 glomerulopathy is a rare disease, characterized by an abnormal activation of the complement’s alternative pathway that leads to the accumulation of the C3 component in the kidney. The disease recurs in more than half of kidney transplant recipients, with a significant impact on graft survival. Recurrence of the primary disease represents the second cause of graft loss after organ rejection. In C3 glomerulopathy, there are several risk factors which can promote a recurrence during transplantation, such as delayed graft function, infection and monoclonal gammopathy. All these events can trigger the alternative complement pathway. In this review, we summarize the impact of C3 glomerulopathy on kidney grafts and present the latest treatment options. The most widely used treatments for the disease include corticosteroids and mycophenolate mofetil, which are already used chronically by kidney transplant recipients; thus, additional treatments for C3 glomerulopathy are required. Currently, several studies using anti-complement drugs (i.e., eculizumab, Ravalizumab, avacopan) for C3 glomerulopathy in kidney transplant patients are ongoing with encouraging results.

## 1. Introduction

Kidney transplantation is the treatment of choice for patients with end-stage kidney disease (ESKD), not only improving their quality of life but also reducing the risk of death from all causes [1,2]. However, there are many complications that can arise after a kidney transplant, including infections, rejection and disease recurrence [3,4,5,6], that can impact graft outcomes and survival. 

**Figure 1:**
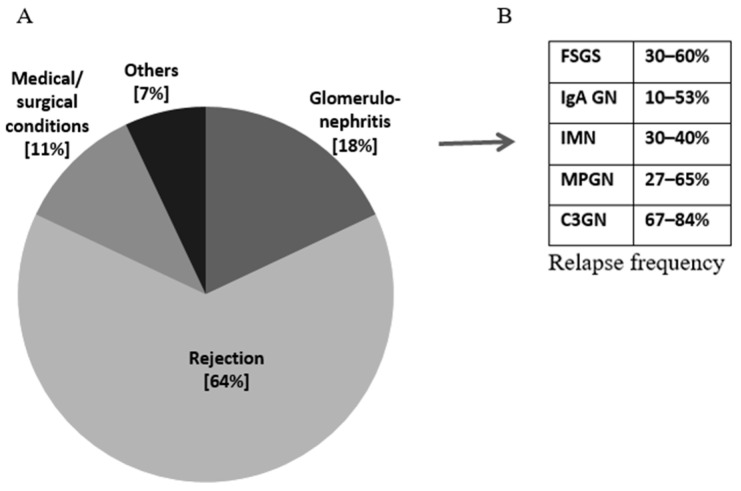
(**A**) Main causes of graft loss. (**B**) Relapse frequency of glomerular disease [9,10,11,12,13,14].

Currently, glomerular diseases represent the second cause of graft loss after rejection (Figure 1A), with a significant impact on long-term graft survival [4,5,6,7,8]. They can be classified as de novo, recurrent or donor-derived. Several studies have investigated which diseases have a greater risk of recurrence [6], their impact on graft survival and possible treatments [9,10,11,12,13,14]. As shown in Figure 1B, C3 glomerulopathy (C3GN) is the glomerulonephritis with the highest frequency of recurrence (67–84%), followed by focal segmental glomerulosclerosis (FSGS) (30–60%), idiopathic membranous nephropathy (IMN) (30–40%), membranoproliferative GN (MPGN) (27–65%) and IgA nephritis (10–53%) [9,10,11,12,13,14]. 

In this review, we will summarize the impact of C3 glomerulopathy on kidney grafts and present the latest treatment options. 

## 2. C3 Glomerulopathy: Pathogenesis and Clinical Features

C3GN is a characterized by an abnormal activation of the complement alternative pathway that leads to the accumulation of C3 components in the glomeruli. The definition of C3GN is recent, as in the past it was defined as membranoproliferative glomerulonephritis (MPGN) type II and included both dense deposit disease (DDD) and C3GN as a single entity [15].

The pathogenesis of C3GN can involve genetic mutations of parts of several components of the complement system (i.e., Factor B [FB], Factor H [FH], Factor I [FI], Complement factor H-related proteins [CFHR], C3) or the development of autoantibodies (i.e., anti-FH, anti-FB, C3Nef, anti-C3 antibodies) leading to the activation of the complement alternative pathway [16]. The diagnosis is biopsy-proven and is based on histological patterns of MPGN in the light microscopy (i.e., lobulation of the glomerulus, moderate absorption of the capillary wall, obliteration of the capillary lumens, increase in the mesangial matrix), the presence of subendothelial deposits in electron microscopy and the presence of C3 deposits at the immunofluorescence with no or minimal Ig deposits.

C3GN recurs in more than 50% of kidney transplantations (KTs), in most cases during the first year, and generally with a slow progression [10,11,12]. However, it is important to distinguish C3GN from DDD. These two diseases present similar histological features and share common pathogenic mechanisms but differ greatly in prognosis and risk of recurrence after KTs (70% for C3GN and 80% for DDD, respectively) [7]. Even if both can impact graft survival [17], C3GN is generally more aggressive [7,18], although Remu et al. found a higher prevalence of graft loss in KT patients with DDD recurrence compared to those with C3GN recurrence [19].

Caravaca et al. analyzed the incidence of MPGN recurrence in 220 kidney graft recipients between 1981 and 2021 [20]. MPGN recurred in 25% of the grafts, and complement-mediated MPGN showed a higher recurrence [62%], with a median time of 14 months and a worse impact on graft survival, with 35% of kidney failures (KFs) [20]. In line with these observations, another study showed that in patients with MPGN as a primary disease, the presence of nephrotic syndrome and complement consumption in a native kidney predicts a more severe disease. In this analysis, the impact of MPGN was lower compared to the literature (between 11.8% and 18.9%), probably due to the maintenance therapy with corticosteroids after KTs [21].

The impact of C3GN recurrence on graft survival depends on its pathogenesis. C3GN due to a mutation of CFHR5 nephropathy is often associated with a favorable long-term outcome [22]. Frangou et al. retrospectively analyzed 17 KT patients with ESRD caused by an internal duplication of exons 2–3 within the CFHR5 gene. They found a recurrence of 70.6% and a favorable long-term outcome [23]. Neetika et al. described a clinical case of a 51-year-old man with ESKD due to C3GN that was secondary to mutations in FH and FI who rapidly developed C3GN recurrence after a KT [24]. In this case, severe ischemia-reperfusion injury (IRI) and delayed graft function (DGF), activating the complement system, may have promoted the development of C3GN [24]. Furthermore, other diseases, such as atypical haemolytic uremic syndrome (aHUS), which is characterized by complement abnormalities, can affect the graft. A French group reported two cases of aHUS associated with CFH deficiency that developed glomerulonephritis with C3 deposits after KTs [25].

In consideration of the importance that C3GN might have on kidney outcomes, several studies have been conducted to correlate clinical and histologic parameters with renal outcomes [26,27]. Lomax-Browne et al. analyzed the kidney biopsy features of patients with C3GN and idiopathic Ig-associated 98 MPGN to produce a prognostic score [26]. Using a multivariate analysis, they demonstrated a negative association between eGFR, crescents, interstitial inflammation and interstitial fibrosis/tubular atrophy (IFTA) and a positive association between proteinuria and endocapillary hypercellularity and glomerular basement membrane (GBM) double contours. In addition, by using a composite outcome, they demonstrated a negative prognostic significance for cellular/fibrocellular crescents and IFTA scores [26].

As is well documented, C3 deposits worsen graft outcomes. However, several factors can influence graft survival, such as the development of transplant glomerulopathy (TG). The latter is defined by histologic features including glomerular basement membrane reduplication shown by light microscopy or by the absence of immune complex deposits in electron microscopy, with a cg score ≥ 1a based on the Banff score 2017 [28]. TG is caused by repeated endothelial cell injuries and not by a specific clinic pathologic entity, with a prevalence of 5–20% in KTs [29]. It reduces graft survival and can be caused by antibody-mediated rejection (ABMR) and not only HLA antibody-related T cell-mediated rejection, thrombotic microangiopathies (TMAs) and hepatitis C virus (HCV) infection [30]. In 2019, Panzer et al. demonstrated that patients with TG and glomerular C3 deposition showed a higher risk of allograft failure compared to patients with TG and no C3 deposition, suggesting that complement activation and C3 deposition are independent risk factors for graft loss [31]. 

## 3. C3 Glomerulopathy and Infections

C3GN presents histological features similar to post-infectious GN (PIGN), and for this reason, differential diagnosis may be challenging. Furthermore, C3GN can be preceded by an infection as well. However, differential diagnoses are important because they differ both in prognosis and risk of recurrence [32]. PIGN is frequent in children and most commonly caused by a Group A β-hemolytic Streptococcus infection. Additionally, in PIGN, there is an activation of the alternative complement system that is mediated by immune complexes; this activation attracts inflammatory cells. Furthermore, during the acute phase, there is an increase in autoantibodies against FB that leads to low plasma C3 levels and increased levels of C5b-9 [32]. In a retrospective study, Chauvet et al. analyzed 34 children with acute PIGN and low C3 levels. At the onset, they found higher levels of FB autoantibodies in children with PIGN versus C3GN; these autoantibodies are transient and correlate with C3 and C5b-9 plasma levels [33].

Currently, there are no specific markers that allow us to differentiate these two diseases, C3GN and PIGN, but it can be useful to evaluate kidney functional recovery: an incomplete recovery of kidney function, associated with persistence of low serum C3 levels for 8–12 weeks after the initial diagnosis, can be considered a sign of C3GN [34,35].

Usually, complement disorders, such as HUS and C3GN, require an external trigger, such as an infection, to manifest, and it is important to underline that transplant patients present a high risk of infection due to the immunosuppressive therapy. The association between C3GN recurrence and viral infections has been described with cytomegalovirus infections that induce a type I MPGN, mimicking a recurrence of the primary renal disease. In these cases, an antiviral therapy (valgancyclovir), rather than an increase in immunosuppressive therapy, is required [36]. During the coronavirus pandemic, several studies showed that, based on ACE-2 receptor expression [37], SARS-CoV-2 infection can affect the kidneys [38,39,40], causing direct and indirect damage [41] that is characterized by the onset of proteinuria and/or acute kidney injury and may lead to a complement activation. All complement pathways can be directly or indirectly activated during SARS-CoV-2 infection, with a worsening of renal function [42]. Pfister et al. have analyzed the kidney biopsies of COVID-19 patients, showing the activation of all complement pathways, in particular the lectin pathway in the peritubular capillaries, which is a classical pathway in the renal arteries and the alternative pathway at the tubular level [42].

Wen et al. described a case of C3GN and thrombotic microangiopathy in a young male with a KT that developed after a pulmonary infection [43]. Genetic analysis revealed two missense variations in the heterozygous form of the complement FI gene, with plasma FI levels in normal range. They hypothesized that infections can act as a trigger for the development of C3GN and thrombotic microangiopathy in genetically susceptible patients [43].

Finally, as C3GN recurrence requires an immunosuppressive treatment that may lead to viral replication, careful surveillance is required. Jeong-Hoon Lim et al. observed that the increased immunosuppressive drug dosage of a 33-year-old male patient with recurrent C3GN following a KT induced an increase in Poliomavirus BK (BKV) replication with a documented new-onset BKV nephropathy and decreased C3GN activity [44].

## 4. Treatment of C3 Glomerulopathy Recurrence

Significant improvements have been made in understanding the pathophysiology of complement-mediated diseases, and with this, the number of therapies and treatments available for native kidneys is rapidly increasing [45]. On the other hand, despite the high recurrence of C3GN and its impact on graft survival, there are no guidelines about its treatment in kidney graft recipients, which is also due to the fact that these patients already receive immunosuppressive therapy, which is usually characterized by a triple-drug regimen, including corticosteroids, calcineurin inhibitors and mycophenolate mofetil (MMF) and that MMF and corticosteroids are the most-used drugs for the treatment of C3GN in the native kidneys [46]. A Spanish group analyzed the outcomes of 97 patients with diagnoses of C3GN (81 patients) and DDD (16 patients) based on the disease pathogenesis, autoantibodies or complement abnormalities and who were treated with MMF and corticosteroids, eculizumab, other immunosuppressive therapies or conservative management. They demonstrated that treatment with MMF and corticosteroids resulted in a significantly better clinical outcome compared to the other approaches [46].

**Figure 2:**
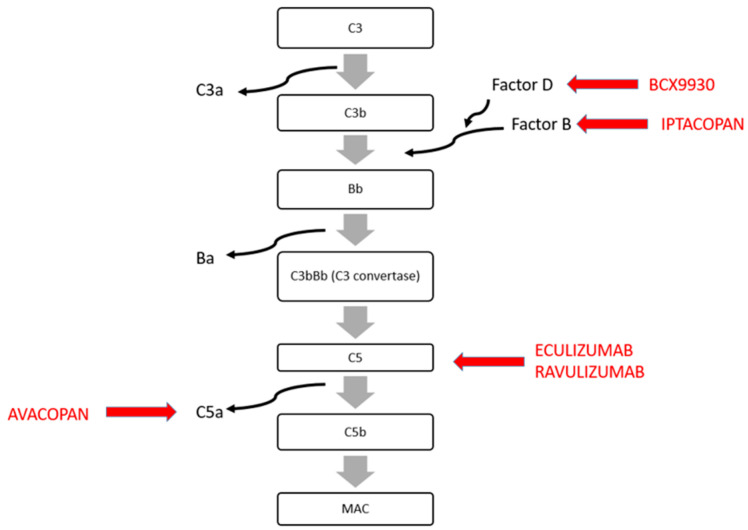
Alternative pathway of complement and anti-complement drugs (red).

Recently, several complement-targeted therapies have been proposed as a treatment for C3GN. The mechanisms of action of these new drugs are illustrated in Figure 2 and summarized in Table 1. Briefly, the complement system can be activated by interactions with the antigen–antibody complex (the classical pathway), with microbial surfaces (the lectin pathway) or by the alternative complement pathway. The alternative complement pathway is important in signal amplification through the formation of C3 convertase, which cleaves C3 and C5 in C3a and C3b and C5a and C5b, respectively (Figure 2). C3a and C5a are pro-inflammatory molecules, while C3b plus FB [Bb] form the C5 convertase. C5b is part of the membrane attack complex that leads to cells lysis (Figure 2).

Another important aspect of the alternative complement pathway is that its regulation is mediated by complement inhibitory factors, including FH, FI and MCP. In particular, FH is the most important regulator of the alternative pathway, blocking the formation of the C3 convertase, promoting its dissociation and cleaving C3b to iC3b. In C3GN, as well as in HUS, there is a dysregulation of the alternative complement pathway, leading to its hyper-activation.

Based on the role of the alternative complement in the pathogenesis of C3GN, several anti-complement-targeted therapies, such as anti-C5 therapies [Ravalizumab, avacopan, Crovalimab, Nomacopan, etc.], Iptacopan and BCX9930 are currently being used in clinical trials [47,48,49,50,51,52,53]. Here, we will discuss the possible use of eculizumab and other anti-complement drugs for the treatment of C3GN and its recurrence in kidney transplants [47,48,54,55].

Eculizumab is the first humanized anti-C5 monoclonal antibody that binds C5 to prevent the formation of the MAC, blocking the damage mediated by the terminal part of the complement cascade. It was first introduced for the treatment of paroxysmal nocturnal hemoglobinuria [49] and, subsequently, it was used for treatment of aHUS, with important results in improving renal function [50]. Several kidney diseases, including IgA nephropathy, C3GN and ANCA-associated vasculitis, are characterized by an increased activation of the alternative complement pathway, leading to complement-mediated kidney damage. Gurkan et al. analyzed the efficacy of eculizumab in a 21-year-old patient with C3GN and found that eculizumab partially prevented C3GN progression, with a clinical improvement of renal function, along with a reduction in proteinuria and an improvement in C3 serum levels [47]. However, this was not mirrored in the allograft biopsies that revealed damage progression and increased chronic damage [47].

Also, Kaartinen et al. presented a clinical case of a kidney graft recipient with recurrent C3GN that was treated with eculizumab [56]. The patient seemed to benefit from the treatment, with a reduction in p-SC5b-9 plasma and no chronic damage at the graft biopsy, but a follow-up biopsy showed progressive damage [56]. 

Subsequently, Regunathan-Shenk et al. analyzed the outcomes of 19 patients with C3 glomerulopathy on their kidney grafts (12 C3GN and 7 DDD), who had a recurrence rate above 80% [19]. They analyzed different therapeutic approaches, including rituximab, eculizumab and increased MMF doses, and reported that eculizumab therapy did not improve graft function. Furthermore, complement pathway abnormalities and genetic risk factors were not associated with clinical responses to eculizumab. On the other hand, all patients treated with rituximab started dialysis therapy [19]. 

Suarez et al. reviewed twelve studies that included seven cohort studies and five case series, comparing patients’ treatments with eculizumab, rituximab, plasmapheresis and no therapy [48]. The total number of patients with diagnosis of post-transplant C3GN was 122. They observed that eculizumab reduced graft losses compared to rituximab and plasmapheresis (33% eculizumab, 42% plasmapheresis, 81% rituximab). The response to eculizumab was related to the initial soluble C5b-9 levels. Indeed, patients with higher levels of C5b-9 showed a better treatment response. Sixty-six patients with a stable allograft function that did not receive any treatment had a graft loss of 40% (32% with C3GN and 53% with DDD) and a lower incidence of kidney allograft injury and proteinuria compared with treated patients [48].

Naseer et al. described the use of Repository Corticotropin (Acthar) in association with eculizumab for the treatment of de novo C3GN in a 48-year-old African American male with a kidney transplantation [57]. The patient showed a resolution of proteinuria within 3 months of therapy with eculizumab and Repository Corticotropin. Interruption of the treatment induced a recurrence of proteinuria, which was firstly reduced with Acthar therapy. Complete proteinuria remission was observed after also restarting eculizumab treatment. It is well known that Repository Corticotropin induces proteinuria remission in nephrotic syndromes through its bind with the melanocortin 1 receptor, which is normally present on podocytes and overexpressed during podocyte injuries [58,59]. For this reason, Repository Corticotropin is very useful in primary podocytopathy and probably also in reducing proteinuria in C3GN. 

Ravulizumab is a humanized monoclonal antibody that has the same targets as eculizumab (the C5 protein epitope); thus, it inhibits the cleavage of C5 into C5a and C5b. Differing from eculizumab, Ravalizumab has a longer half-life, and this allows an infusion rate of every 8 weeks instead of the 2 weeks of eculizumab. Phase III studies (ALXN1210-aHUS-311) have shown that Ravulizumab could induce a complete TMA response, an improvement in renal function and dialysis weaning in more than half of patients. Currently, Ravalizumab is approved by the Food and Drug Administration (FDA) for aHUS treatment, and it is under investigation for the treatment of secondary thrombotic microangiopathies, IgA nephropathy and lupus nephritis [60,61,62,63]. 

Avacopan (CCX168) is an anti-C5 drug that acts by blocking the binding between C5a and its receptor 1, C5aR1. Thus, it selectively inhibits the terminal cascade of the complement. Avacopan is orally administered, and it is currently approved in Europe for the treatment of adult patients with severe active granulomatosis with polyangiitis or microscopic polyangiitis. 

Animal model studies suggest that in C3GN, the inhibition of C5a is more important than the inhibition of C5b-9 [64,65]. ACCOLADE is a Phase II study with the aim to evaluate the effects of avacopan treatment on patients with C3GN with or without a KT (Clinical Trial NCT03301467). ACCOLADE is randomized, double-blind, placebo-controlled study that enrolled 57 patients with a follow-up period of 26 weeks [placebo vs. avacopan]. After this period, all patients received avacopan for 26 weeks. The primary endpoint of this trail was a change in the histologic index of disease activity. Zotta et al. report a case of a 11-year-old female child with a histological diagnosis of C3GN and no genetic mutations identified [52]. She was initially treated with i.v. pulses of methylprednisolone and then oral prednisone, in addition to MMF and ACE inhibitor. Due to the relapse of proteinuria, cyclosporine A was also introduced. The patient did not achieve remission and she was started on avacopan, maintaining her therapy with MMF, cyclosporin and ACE inhibitor, with a reduction in proteinuria. When avacopan or cyclosporin was discontinued, she experienced a relapse of proteinuria. For this reason, both avacopan and cyclosporin were rapidly reintroduced with a good response. After 12 months of remission, she discontinued MMF, maintaining a remission of C3GN [52].

Another approach to slow down complement activation through the alternative pathway includes the inhibition of Factor D (FD). In the alternative complement pathway, FD cleaves FB bound to C3b into Ba and Bb, allowing the formation of the C3bBb complex that is the C3 convertase of the alternative pathway. BCX9930 is an oral Factor D inhibitor that is currently being studied for the treatment of C3GN, IgA nephropathy and MN (NCT05162066). This is an open-label, multicenter, non-randomized study in which BCX9930 is administered to adult patients for 52 weeks. The aim is to evaluate the safety and therapeutic potential of this drug. In total, 14 patients with C3GN have been enrolled (NCT05162066).

Finally, Iptacopan is an oral selective inhibitor of complement FB. APPEAR-C3G (NCT04817618) was a randomized, double-blind and placebo-controlled Phase III study. The aim was to evaluate the efficacy and safety of Iptacopan in 68 C3G patients. In the first 6 months, patients received a placebo or Iptacopan, and then all patients received Iptacopan. The study showed a significant reduction in C3 protein deposits in kidney biopsies compared to the baseline both in C3GN-native kidneys and in C3GN that returned following a kidney transplant. Furthermore, Iptacopan induced a reduction in proteinuria and a normalization of serum C3 levels [53]. Currently, a clinical trial to evaluate the long-term effect of Iptacopan is ongoing (NCT03955445).

**Table 1:** Novel anti-complement drugs for C3 glomerulopathy (C3GN). PNH: paroxysmal nocturnal hemoglobinuria; aHUS: atypical hemolytic uremic syndrome; IgAN: IgA nephritis; LN: lupus nephritis; GPA: granulomatosis polyangiitis, MPA: microscopic polyangiitis; anti-neutrophil cytoplasmic autoantibody (ANCA)-associated vasculitis; MN: membranous nephropathy.

Treatment	Target	Mechanism of Action	Os/Ev	Status	References
Eculizumab	Anti-C5 monoclonal antibody	Block the formation of MAC	Ev (every 2 weeks)	Approved for PNH, aHUS	Regunathan-Shenk R. et al., 2019 [19] Gurkan et al., 2013 [47] Gonzalez Suarez et al., 2020 [48] Kaartinen et al., 2018 [56] Hillmen et al., 2020 [49] Fakhouri et al., 2020 [50] Naseer et al., 2022 [57]
Ravalizumab	Anti-C5 humanized monoclonal antibody	Block the formation of MAC	Ev (every 8 weeks)	Approved for aHUS/Ongoing IgAN, LN	ALXN1210-aHUS-311 60–63
Avacopan (CCX168)	C5a inhibitor	Block the binding of C5a-C5Ar1	Os	Approved for ANCA-associated vasculitis/Ongoing for GPA, MPA	ACCOLADE trial Zotta et al., 2023 [52]
Iptacopan	Selective Factor B inhibitor	Block the formation of C3 convertase	Os	Approved for PNH/Ongoing for complement disease	APPEAR-C3G
BCX9930	Factor D inhibitor	Block the formation of C3 convertase	Os	Ongoing for C3GN, IgAN, MN	NCT05162066

## 5. C3 Glomerulopathy and Monoclonal Gammopathy: Description and Treatment

Another important aspect of C3GN is its association with monoclonal gammopathy [66,67,68], which includes all diseases characterized by the presence of a monoclonal protein, namely multiple myeloma, Waldenström macroglobulinemia, amyloidosis and monoclonal gammopathy of renal significance. Monoclonal protein activates the complement cascade [33,69,70,71], causing proliferative glomerulonephritis [70,72]. In 1992, Meri S et al. found that monoclonal immunoglobulin lambda light chain dimer is able to interact with complement FH [70]. This interaction inhibits the activity of FH and leads to the activation of the alternative complement pathway. In addition, it differs from the nephritic factor in that the latter is unable to stabilize the C3bBb enzyme [70]. In the literature, several clinical cases have been described [73,74].

Mayo Clinic analyses found a higher prevalence of monoclonal immunoglobulins in older C3GN patients. However, not all patients had a low C3 levels, but on the other hand, they had more C3Nef or other autoantibodies against complement-regulating proteins [75]. Lloyd et al. described similar results: they analyzed 12 patients (≥50 years old) with C3GN and found paraproteinemia in 83% of patients with a poor renal prognosis [76]. 

Occasionally, this association may not be evident in native kidneys, but it develops after KTs or it can recur early on the graft [77,78,79]. Ruiz-Fuentes et al. presented a case of an older patient with ESKD due to unknown origins and monoclonal IgGl gammopathy with a low risk of progression who underwent a a KT [74]. In the graft, he developed C3GN, with no response to eculizumab treatment and need of dialysis [74]. 

Serra et al. described three cases of C3GN in KT patients with de novo monoclonal gammopathy [80]. The patients developed MGRS between 6 and 10 years after transplantation and started dialysis 2 to 15 months after the diagnosis of MG. Only in one case was rituximab used for treatment (it was an IgM kappa monoclonal component, and there was the presence of anti-factor H antibodies). 

In a cohort of four Caucasians without hematologic malignancy, a recurrence of disease was diagnosed in a median time of 3.8 months after transplantation, with a good response to high-dose prednisone plus rituximab or plus cyclophosphamide and stabilization of the disease [81].

Treatment of C3GN associated to monoclonal gammopathy includes the use of chemotherapy, i.e., bortezomib, cyclophosphamide, dexamethasone and rituximab, targeting the underlying B-cell clone. 

In 2017, Chauvet S et al. analyzed the renal outcomes of 50 patients with monoclonal gammopathy in a retrospective multicenter study, including different treatment regimens, namely immunosuppressive therapy [82], chemotherapy or symptomatic measures alone. The authors described higher renal survival of patients treated with chemotherapy or immunosuppressive therapy compared to conservative treatment. Noteworthily, the most important factor that predicted renal outcomes was the hematological response to treatment that correlated with a better renal prognosis. Furthermore, four patients underwent KTs (with no hematological response at the time of transplantation), and all patients had a recurrence of the disease in 1 year, with one case of graft loss [82]. 

Heybeli et al. presented a case series of patients with monoclonal gammopathy who underwent KTs over a period of 29 years; among these, 5 patients out of 30 were affected by C3G-MG. All patients (both previously treated and not) developed a recurrence of the disease, with a median time of 70 days after transplantation, and only a patient with a complete hematological remission after recurrence had a better prognosis [73]. 

Moog et al. reported a clinical case of an early recurrence of monoclonal gammopathy-related C3GN on a kidney graft (2 months after the KT) that showed a good response to eculizumab–bortezomib treatment in association with standard immunosuppression [83]. 

Rituximab treatment has been analyzed in patients with MPGN and monoclonal IgG deposits [84,85]. A good renal response has been observed in patients without hematologic malignancy who were treated with rituximab, with less adverse effects compared to chemotherapy. However, currently, the use of rituximab for the treatment of C3GN is not recommended [86].

C3GN associated to MG recurs early on grafts and is associated with poor graft outcomes [77]. For this reason, a correct hematologic diagnosis, its treatment before transplantation and prevention are essential. 

## 6. Conclusions

C3GN is a rare disease with an important impact on kidney outcomes, both for native kidneys and kidney grafts. Over time, important progress has been made in understanding its pathogenesis, its risk factors and its treatment. As previously stated, kidney transplantation improves quality of life, reduces comorbidities and, in particular, it reduces mortality compared to dialysis. However, recurrent diseases have a negative impact on graft survival and, unfortunately, are common in kidney transplant patients. As stated, C3GN is characterized by a high rate of recurrence, with 50–70% frequency during the first year of a kidney transplant. After transplantation, several risk factors are associated with the development of C3GN, such as delayed graft function, infections and neoplasia, including monoclonal gammopathies. All these events may activate the alternative complement pathway, which is necessary for the development of the disease. In the last years, several studies and trials have investigated the best treatment for C3GN in transplanted kidneys, with particular attention given to the disease pathogenesis. Several trials based on the use of drugs targeting complement factors are ongoing with encouraging results. Despite these efforts, neither the latest core curriculum of the management of kidney transplant recipients (2019) [87] nor the KDIGO guidelines give guidance on how to treat relapsed or de novo C3GN in transplant patients. Furthermore, when C3GN is associated to monoclonal gammopathies, it is important to treat the hematological disease before transplantation since hematological response is associated with a better renal survival and a lower risk of early graft recurrence. Although in recent years, probably thanks to new potential drugs, attention to C3GN has increased [88,89,90], further studies are needed to better understand the pathogenesis, course and potential therapy for C3GN both on native kidneys and on transplanted kidneys.
